# Pharmacogenomics in Personalized Medicine and Drug Metabolism 

**DOI:** 10.1155/2014/897963

**Published:** 2014-07-17

**Authors:** Wei-Chiao Chang

**Affiliations:** ^1^Department of Clinical Pharmacy, School of Pharmacy, Taipei Medical University, No. 250, Wuxing Street, Xinyi District, Taipei 110, Taiwan; ^2^Department of Pharmacy, Taipei Medical University-Wan Fang Hospital, No. 111, Section 3, Xinglong Road, Wenshan District, Taipei 116, Taiwan; ^3^Master Program for Clinical Pharmacogenomics and Pharmacoproteomics, School of Pharmacy, Taipei Medical University, No. 250, Wuxing Street, Xinyi District, Taipei 110, Taiwan; ^4^Graduate Institute of Clinical Medicine, College of Medicine, Kaohsiung Medical University, No. 100, Shiquan 1st Road, Sanmin District, Kaohsiung 807, Taiwan

Enormous progress in human genetic studies has been made in the past decade. With the international research collaboration and advances in genome sequencing techniques, understanding of human genome and disease pathogenesis has dramatically increased. In particular, the information from human genome gives us more ideas to understand disease susceptibility and drug responses. Personalized medicine and pharmacogenomics are to determine unique molecular characteristics between the individuals and to apply this genetic information to diagnose an individual's disease accurately, select better treatments, and reduce possible drug adverse reactions.

This special section contains eleven articles. S.-I. Hung et al. carefully reviewed recent findings in genetic variations and adverse drug reactions. R.-H. Wong et al. described their work that indicated that pesticide exposed individuals with susceptible MDR1-129 genotypes may have increased risk of DNA damage. M.-S. Wu et al. reported a genetic polymorphism in ORAI1 calcium channel that associated with elevated serum calcium levels in chronic kidney disease patients. Q. Jiang et al. showed that ESR1 gene is considerably associated with knee osteoarthritis etiology in the Chinese Han population. Y. Wang et al. identified that polymorphisms in interleukin-4 (IL-4) and interleukin-6 (IL-6) are associated with increased risk of rheumatoid arthritis. Using bioinformatics, E. Y. Chuang's group and C.-H. Yang's group successfully identify more genes linked with breast cancer and Alzheimer's disease. J.-Y. Wang et al. reported that chip including DPYD, TYMS, TYMP, TK1, and TK2 genes is a potential tool to predict responses in locally advanced rectal cancer patients treated with fluoropyrimidine-based chemoradiotherapy. J.-H. Li et al. provided convincing evidence that CYP2B6 785G allele and ABCB1 2677T allele have positive effects on the methadone plasma concentrations. J. Wei et al.'s work indicated that PDZ-binding motif (TAZ) is a potential marker that is overexpressed in signet ring cell carcinoma. In addition, K.-S. Hung et al. reported new clinical applications to improve motor function after traumatic brain injury. Y.-W. Cheng et al. identified a new compound (TW01003) with potent anticancer and antiangiogenesis activities.

In conclusion, the field of genomic medicine has largely advanced in a relatively short time. With the new techniques now available, new findings will be revealed in the future.

*Wei-Chiao Chang*

